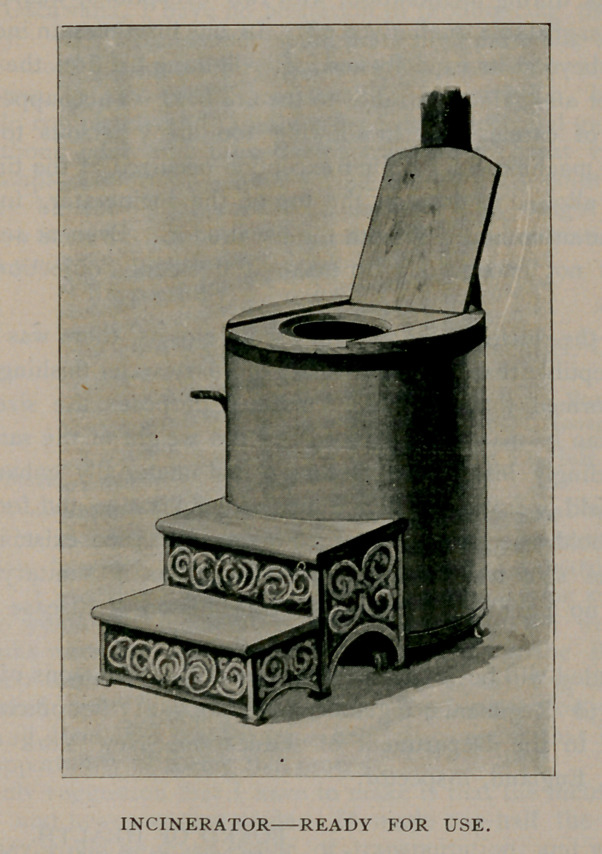# The Incineration of Garbage and Excrement at Military Camps

**Published:** 1900-02

**Authors:** W. G. Bissell

**Affiliations:** Buffalo, N. Y.


### THE INCINERATION OF GARBAGE AND EXCREMENT AT MILITARY CAMPS.

A REPORT TO THE HEALTH COMMISSIONER OF BUFFALO.

By W. G. BISSELL, M. D., Buffalo, N. Y.

I HAVE the honor to submit the following report pertaining to the incineration of garbage and excrement, carried on under my direction at the State Camp of Military Instruction during the last season.

May 22, 1899, the following circular letter was sent to many of the leading sanitarians of the country :

*Dear Sir.*—I have been informed by the New York State National Guard authorities, that an opportunity is to be provided in the near future for a series of experiments to be conducted under my direction, pertaining to the practical application of methods of incineration for excrement and garbage, to be used by military forces in field service.

Should you have ideas on this subject, I will be greatly honored if you will acquaint me with same and will endeavor to put in practical operation any beneficial suggestions that may be offered. In the final report on this subject, special credit will be given those persons offering suggestions that prove, in this connection, of value.

Respectfully,

WILLIAM G. BISSELL, M.D.,

Bacteriologist, Department of Health, Buffalo, N. Y.

A large number of replies were received but, with few exceptions, he writers did not confine themselves to the subject matter, but dwelt more particularly upon the urgent necessity for a competent system for the disposal of excrement by military forces.

A communication from Dr. Albert L. Gihon, Medical Director of the United States Navy (retired), was the single exception where reference was made to the use of the instruments with which I was directed to conduct tests.

Dr. Gihon states that "an instrument I saw in Buffalo a couple of years or more ago seemed to me to possess all the requisites and to be particularly adapted for military field service, being transportable, effective and inexpensive."

Communications from Dr. U. O. B. Wingate, of Milwaukee, and Dr. Benjamin Lee, of Philadelphia, I also submit as being of particular interest.

STATE OF WISCONSIN, STATE BOARD OF HEALTH,
EXECUTIVE OFFICE, MILWAUKEE, May 27, 1899.

DR. WM. G. BISSELL, Bacteriologist, Department of Health, Buffalo, N. Y.

*Dear Doctor.*—I am in receipt of yours of the 23d inst., in which you ask if I have any ideas relative to the matter of the incineration of excrement and garbage, and that you would be grateful if I would acquaint you with the same. In reply, permit me to state that in what experience I have had in this line, I have never yet seen a crematory that was able to consume all the gases generated by this material.

I presume that the process of incineration has been improved upon to that extent that this can be done now, but if so I would like very much to know it. This has been the principal trouble that I have met with in attempting to burn night-soil, or more especially garbage.

There are certain acid gases which it has seemed to me are incumbustible by any degree of heat that I have seen used in the past. I am unable to state to you what these gases are. All I know is that they produce an intolerable stench whenever incineration has been used in my experience in times past. I have not, however, witnessed the operation of more recent apparatus used for this purpose.

I should be highly honored if you can give me any light on this subject of the combustibility of certain gases that are generated by certain degrees of heat from this material. Thanking you in advance for any information you may give me, I remain,

Very truly yours,
U. O. B. WINGATE, Secretary.

COMMONWEALTH OF PENNSYLVANIA, STATE BOARD OF HEALTH,
EXECUTIVE OFFICE, 1420 CHESTNUT STREET.
PHILADELPHIA, May 25, 1899.

DR. WM. G. BISSELL, Bacteriologist, Department of Health, Buffalo, N. Y.

*Dear Sir.*—Replying to your favor of the 23d inst., I would say that recent inquiries have led me to the conclusion that there is not at present, any apparatus or contrivance for incineration of excrement and garbage which would be of use in field service.

I am glad to know that the New York National Guard authorities are taking up the question, as I feel it to be one of great importance. There would, of course, be no difficulty in constructing a cremation furnace for a permanent camp.

My own view is that if it were necessary to leave off a few cannon from the equipment of a corps and provide instead portable crematories which, I think, could be devised, the gain would be very great. But it seems almost impossible to make purely military authorities understand the importance of preserving the health of the soldier.

Yours very truly,

BENJAMIN LEE, Secretary.

The instruments experimented with by myself were constructed by the International Garbage and Crematory Company of Buffalo, N.Y.,(p. 503) and,so far as I am, at this time, able to ascertain,are the only strictly portable incinerators made in the United States, adaptable for military camps and field purposes.

The accompanying cut (p. 502) plainly shows the interior construction,of which "A" is the upper space between the pot or receptacle "B" and the top of the casing; "C" is the fire-box, and "D" is the draft passage; "E" is a passage leading down from the upper space "A" into the draft passage "D" and "F" is an opening leading from the fire-box up into the draft passage "D". A deflecting wall "G" is located midway between the passage "E" and opening "F." The odors from the material in the pot or receptacle are drawn down through the passage "E" by the draft, as indicated by the arrows, and pass through the draft passage "D" into chimney or smoke pipe "H," and the products of combustion from the fire-box "C" pass up through the opening "F" into the same draft passage "D", mingling therein with the odors and gases from the upper space "A", which are destroyed before passing into pipe "H."

For use, the incinerators were placed in a convenient location, and pieces of pipe three feet in height were attached to portions, as shown in diagram at "H." At the time of incineration, the wooden seat supplied with each instrument was replaced by an iron cover. The draft near the smoke-pipe was closed and fire made by using wood as fuel.

June 14, 1899, ten machines accompanied the 2d Battalion of the 23d Regiment on their "march out" from the State Camp to the first camp established by them on land known as the "Croft Farm." This battalion had an aggregate of 268 men present. The incinerators were transported on two ordinary wagons designed for carting purposes, each being drawn by a single team. At the first camp ten incinerators were used. Careful observation as to whether this number supplied the demand for all purposes demonstrated that, at no time, was there occasion for wait. This battalion moved from the first camp on the 15th instant. A detail, under my charge, consisting of three military men and two teamsters completed incineration and loaded five of the instruments in time to catch up with the wagon train. These instruments were ready for transportation in forty-eight minutes after the departure of the troops.

INCINERATOR—SECTIONAL VIEW.

At the second camp (Lake Oscawana) ten machines were used and on the morning of the 16th instant, the excrement contained therein was completely incinerated in about one hour.

June 19, 1899, the first camp on the "Croft Farm" was occupied by the 1st Battalion of the 7th Regiment, and thirteen incinerators were used. This battalion was composed of 430 men, including officers and servants. The troops remained in camp during the day of the 19th until early morning of the 20th inst. On the morning of the 20th inst., incineration was accomplished with all the machines in less than two hours, and ten of the incinerators were transported to the second camp, the same as that formerly occupied by the 23d Regiment. The number of incinerators used at this camp, including those previously left behind, was fourteen. This number entirely supplied the demand for sink purposes, and on the morning of the 21st instant, after a heavy rainfall, and by the use of wood that had become dampened, incineration was accomplished in about two hours.

INCINERATOR—READY FOR USE.

On the afternoon of the 21st instant, the first camp was occupied by the 2d Battalion of the 7th Regiment, comprising 271 men including officers and servants. Thirteen incinerators were used. On the morning of the 22d instant, incineration was completed in less than two hours.

At the second camp of this battalion incineration with twelve machines was accomplished in one hour and thirty-two minutes. Similar results were accomplished, under varying climatic conditions, including wind and rainfall, with other military organisations.

A thorough test was made as to the disposal of garbage and it was found that garbage in any form could be completely incinerated, the length of time depending upon the nature of the material to be destroyed and the amount of moisture present. As to the practicability of the use of these incinerators for the destruction of excrement and garbage for military purposes and household use, I would state that at no time during incineration, with two exceptions, was there any odor or disagreeable smell given off. In one instance, an incinerator was filled beyond its capacity and, after lighting the fire, the contents boiled over and gained entrance to the fire-box. This happening was the result of carelessness in allowing too many persons to use this individual machine. The other exception occurred at the time when urine was allowed to drop on the top of the incinerator, for, when heated, the urine burns off with the usual odor. Even at such times there was not enough odor to make it decidedly objectionable or obnoxious.

When the incinerators were in use as closets, there was no more odor perceptible than occurs with any ordinary water flushing system. I am informed that for purely militaiy purposes the size of the machine can be materially reduced and the weight of the same made correspondingly less, which factors would materially enhance their value in field operations. For camps of mobilisation and for use in the household where a system of sewerage does not exist, incinerators of the size tested supply every want as a sanitary closet. There is no possible chance of the transmisson of disease by their use.

Appended will be found the opinions of the Surgeons of the 1st, 7th and 23d Regiments, together with a copy of the official report submitted to the Department of Education, New York City, by George B. England, inspector.

BOARD OF HEALTH.

JOHN S. WILSON, M. D., Health Officer.

POUGHKEEPSIE, N. Y., July 8, 1899.

DR. WILLIAM G. BISSELL, Buffalo, N. Y.

*Dear Doctor.*—While on duty at state camp for instruction, Peekskill, N. Y., June 24, 1899, I critically examined your incinerator and watched it while in operation. The incineration was complete and odorless. I believe you have solved a difficult problem in sanitation. You will find a large field of application for your apparatus.

Fraternally,

JOHN S. WILSON, Surgeon, 1st Regiment.

MEDICAL DEPARTMENT SEVENTH REGIMENT, N. G., N. Y.
Park Avenue and 66th St.

NEW YORK, June 30, 1899.

DR WILLIAM G. BISSELL, Buffalo, N. Y.

*Sir.*—I have the honor to acknowledge the receipt of your letter of June 28th. In response thereto, it gives me much pleasure to state that from my observation of the incinerators used under your direction in the recent camps of the Seventh Regiment at Croft's Farm and Lake Oscawana, I have no hesitation in pronouncing them most satisfactory. Of course, I understand that these particular incinerators were not built for military purposes, nor was easy and rapid transportation greatly considered in their construction, but they certainly do completely and rapidly reduce excrement and garbage to ashes, without odor and thus demonstrate their great value for military camps, both from a labor-saving and sanitary point of view. Could they be considerably reduced in size and weight and be mounted on some sort of low vehicle for quick transportation, their usefulness would be greatly enhanced.

Respectfully,

CHRIS. J. COLLES,

Assistant Surgeon, 7th Regiment N. G. N..Y.

THE PROSPECT, ON LAKE BOMOSEEN,
HORACE B. ELLIS, Manager.

CASTLETON, VT., July 4, 1899.

DR. W. G. BISSELL, Buffalo, N. Y.

*Sir.*—Your letter of June 28th, asking for my personal opinion in regard to the incinerators which were tried during the march of the 2d Battalion, has just been received, having been forwarded from Brooklyn. I was very much pleased and surprised at the success of the trial to which the incinerators were put. Only ten were used and no sinks were dug, the number was sufficient for the Battalion. During their use there was no perceptible odor or anything that could be considered in any way objectionable. The incineration of excrement was all done after the Battalion had left camp, so that I did not have an opportunity of seeing that process.

The only suggestion that I have to make is that the incinerator is too bulky and heavy as now made. If made one-half the size and weight, they would be much easier of transportation, and would be large enough for the uses intended.

Respectfully,

HENRY L. COCHRAN,

Surgeon, 23d Regiment, N. G. N. Y.

MEDICAL DEPARTMENT,
SEVENTH REGIMENT, N. G., N. Y., PARK AVE. AND 66th ST.

NEW YORK, June 30, 1899.

To DR. W. G. BISSELL, Buffalo, N. Y.

*Sir.*—I have the honor to report that I have observed carefully the incinerators in use at Croft's Farm and Lake Oscawana during the Camp of the 2d Battalion of the 7th Regiment, N. G. N. Y. I found that the feces previously deposited in these had been entirely consumed, that the fourteen in use were quite sufficient, that by their use the digging of sinks were made entirely unnecessary, that a possible source of danger to the battalion was absolutely removed, and that the hygienic condition of the camps was therefore notably improved. If the incinerator could be made more readily portable and could be mounted on low wheel trucks, suitably covered and screened, the practical use of the apparatus would be furthered.

Respectfully submitted,

JOHN H. HUDDLESTON,

Assistant Surgeon, 7th Regiment, N. G. N. Y.

DEPARTMENT OF EDUCATION,
CITY OF NEW YORK, BOROUGH OF QUEENS.

June 24, 1899.

Report on a new and improved method for the disposal of excrement and garbage, experiments with which were conducted under the personal supervision of Major Bissell, Surgeon, 74th Regiment, N. G. N. Y.

Major Bissell had consulted either by letter or personally almost every man that could give any information on this very important subject, and after careful study and observation, made the practical test at the State Camp, at Peekskill, N. Y., while the 23d Regiment of Brooklyn was at Camp.

The writer feels in duty bound to make such report, after the kindness of the committee in granting him the privilege to accompany the regiment to camp, and he sincerely hopes the report may contain something of value to the Board of Education in their efforts to solve the problem of sewage disposal, or in other words improve the sanitary conditions of the schools that have the so-called "Smead System" of dry closets. This is a very undesirable subject to converse, but it is nevertheless a very important one, and the Board should be as anxious to obtain the benefit of latest thought, study, experience, and knowledge of practical and scientific men in this line as well as in any other branch of study or training.

Undoubtedly the Board is very well informed regarding the "Smead System," yet, a few words may be necessary for the proper filling of the report. It may be defined as follows: The "Smead System," is a vacuum movement of ventilation of the air in the rooms which is exhausted by an upward current of heated air in a connecting flue or duct. This duct may extend through the vault of the closets, so as to make a direct connection with class-rooms. Of course the current is theoretically from the rooms in question, but like a great many other cases in practice, it does not always work as intended.

Imagine a particular case, which is a fair sample of many schools in the Boroughs of Richmond and Queens. A large vault, after being in use for a period of several months and containing some cubic yards of excrement, a back draft or current occurs as is often the case. Further comment is unnecessary.

The theory of the "Smead System" is correct, but in practice it is far from being satisfactory because it has not been developed on scientific lines. Any further information may be had from the excellent works of Dr. Billings and Professor Carpenter.

### FOR THE DISPOSAL OF EXCREMENT.

The excrement should be received in air and water-tight receptacles. The excrement should be cremated after each individual occupation. There should be no porous material in any part of the apparatus such as brick work, soft wood-work or any complex parts that could not be easily removed and cleaned. The apparatus should be an independent and continuous working arrangement, in no way connected with the system of heating and ventilation. The apparatus should be movable and, of course, have the necessary perfected working parts to ensure the continuous action and rough usage. And the operation so complete that it could be placed in any place without any odor from it. Be so reasonable in price so that every school could procure one, however humble their circumstances. So much for the ideal apparatus. We will now proceed to explain experiments which were conducted at Peekskill on June 14 and 15, 1899.

### THE IMPROVED CREMATORS.

There were ten fixtures taken with the 2d Battalion of the 23d Regiment on their "march-out" to Oscawana Lake, some eleven miles distant from camp, over a very rough and hilly country. This was an admirable place for a practical test of the "cremators," and as Major Stokes, who lead the battalion jokingly said, "Why they won't allow them to smell," showing that the learned men had great confidence in their apparatus.

They look not unlike large cylinder stoves, with smoke pipes extending out of the back and with closet seats. The receptacle was a heavy iron pot which held all of the excrement and urine. Ten were found to be sufficient for over 400 men. The pot was directly over and in communication with the firing chamber. After being in use for a day, the fire was started and the contents dried and rendered combustible, after which the pot was dumped directly into the fire and the remaining matter entirely reduced and rendered harmless. In one hour the cremators had been burned out, sterilised, deodorised, cooled and packed for shipment. This goes to show that such an arrangement as described is practicable. This is a subject that should receive the attention of all thoughtful and public-spirited men.

GEORGE B. ENGLAND,
Inspector.

## Figures and Tables

**Figure f1:**
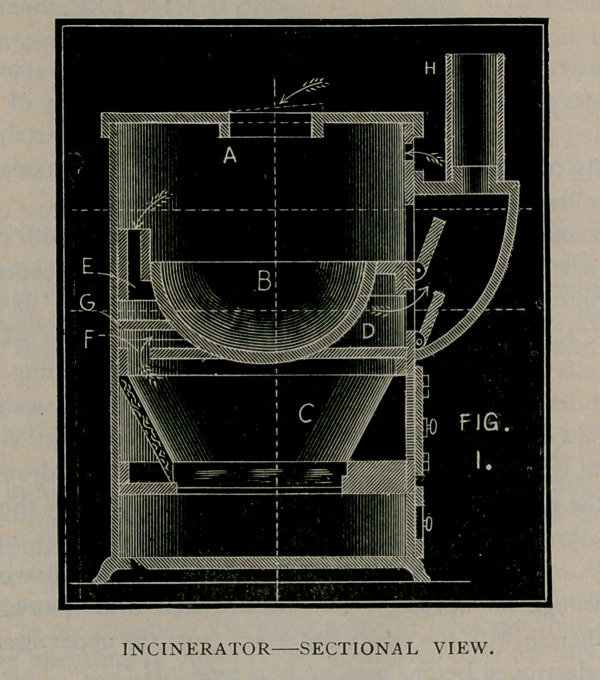


**Figure f2:**